# A novel bi-directional promoter system allows tunable recombinant protein production in *Pichia pastoris*

**DOI:** 10.1186/s12934-017-0768-8

**Published:** 2017-09-13

**Authors:** Vignesh Rajamanickam, Karl Metzger, Christian Schmid, Oliver Spadiut

**Affiliations:** 1 Research Division Biochemical Engineering, Institute of Chemical, Environmental and Biological Engineering, TU Wien, Gumpendorfer Strasse 1a, 1060 Vienna, Austria; 2 Christian Doppler Laboratory for Mechanistic and Physiological Methods for Improved Bioprocesses, Institute of Chemical, Environmental and Biological Engineering, TU Wien, Vienna, Austria; 3 bisy e.U., Wetzawinkel 20A, 8200 Hofstaetten/Raab, Austria

**Keywords:** *Pichia pastoris*, Bi-directional promoter, Bioprocess development, Mixed feed, Tunable recombinant protein production

## Abstract

**Background:**

The methylotrophic yeast *Pichia pastoris* is a well-studied host organism for recombinant protein production, which is usually regulated either by a constitutive promoter (e.g. promoter of glyceraldehyde-3-phosphate dehydrogenase; P_GAP_) or an inducible promoter (e.g. promoter of alcohol oxidase 1; P_AOX1_). Both promoter systems have several advantages and disadvantages; with one of the main disadvantages being their lack of tunability. Various novel promoter systems, which are either inducible or de-repressed, allowing higher degrees of freedom, have been reported. Recently, bi-directional promoter systems in *P. pastoris* with two promoter systems regulating recombinant expression of one or more genes were developed. In this study, we introduce a novel bi-directional promoter system combining a modified catalase promoter system (P_DC_; derepressible and inducible) and the traditional P_AOX1_, allowing tunable recombinant protein production.

**Results:**

We characterized a recombinant *P. pastoris* strain, carrying the novel bi-directional promoter system, during growth and production in three dynamic bioreactor cultivations. We cloned the model enzyme cellobiohydralase downstream of either promoter and applied different feeding strategies to determine the physiological boundaries of the strain. We succeeded in demonstrating tunability of recombinant protein production solely in response to the different feeding strategies and identified a mixed feed regime allowing highest productivity.

**Conclusion:**

In this feasibility study, we present the first controlled bioreactor experiments with a recombinant *P. pastoris* strain carrying a novel bi-directional promotor combination of a catalase promoter variant (P_DC_) and the traditional P_AOX1_. We demonstrated that this bi-directional promoter system allows tunable recombinant protein expression only in response to the available C-sources. This bi-directional promoter system offers a high degree of freedom for bioprocess design and development, making bi-directional promoters in *P. pastoris* highly attractive for recombinant protein production.

## Background

The methylotrophic yeast *Komagataella phaffii*, also known as *Pichia pastoris*, is extensively used as host organism for recombinant protein production (e.g. [1–7]). The main advantages of *P. pastoris* are its fast growth, its ability to use the cheap substrate methanol as sole carbon source, its ability to perform typical eukaryotic post-translational modifications and the possibility of secreting the recombinant product [5, 6, 8–10]. Usually, recombinant protein production in *P. pastoris* is either regulated by a constitutive promoter, like the promoter of glyceraldehyde-3-phosphate dehydrogenase (P_GAP_), or an inducible promoter, like the promoter of alcohol oxidase 1 (P_AOX1_) (e.g. [7, 8, 11, 12]). Both of these prominent promoter systems are characterized by several advantages, but also disadvantages.

The strong, constitutive P_GAP_ allows high product yields in rather short process times [13]. However, cell growth and recombinant protein production are directly linked causing a high metabolic burden for the cells, which might lead to the production of unwanted metabolites or even cell death [14].

On the other hand, biomass formation can be decoupled from recombinant protein production using the inducible P_AOX1_, which is tightly regulated and gives high expression levels [12, 13, 15, 16]. However, the safety aspect for industrial large-scale manufacturing processes with P_AOX1_-driven *P. pastoris* production strains needs particular attention, as the storage of large volumes of hazardous, flammable methanol is highly undesirable. Apart from the fact that methanol makes the fermentation process dangerous and environmentally non-friendly, methanol metabolism leads to great heat evolution and high oxygen consumption, which pose additional challenges for cultivations at large scales.

**Fig. 1:**
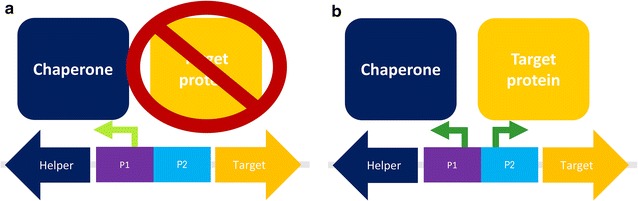
A bi-directional promoter system, where both promoters can be controlled and tuned separately allows **a** the consecutive and **b** the concomitant production of a chaperone and a target protein

Furthermore, a common disadvantage of both promoter systems P_GAP_ and P_AOX1_ is the lack of tunability. It would be highly advantageous to be able to adjust recombinant protein production to different growth conditions and environmental stress to reduce metabolic burden and thus the formation of unwanted metabolites. Besides, it would be highly advantageous to have an expression system in yeast where two recombinant genes can be controlled, regulated and tuned separately. Potential applications of such a system include the balanced co-expression of the individual peptide chains of dimeric proteins, or the consecutive expression of a chaperone followed by the tailored expression of the target product to boost the yield of correctly folded and active product (Fig. 1). Recently, we have used such a system for the recombinant production of the enzyme horseradish peroxidase [17]. However, such a tunable, bi-directional promoter system is not possible using a combination of the promoter systems P_GAP_ and P_AOX1_ due to the aforementioned disadvantages. Thus, a lot of effort is ongoing to identify and investigate novel promoter systems, which are either inducible or de-repressed under limiting conditions [12, 18–26].

In this study, we introduce a novel modified catalase promoter system (P_DC_), which is a 500 bp fragment of the DNA upstream of the peroxisomal catalase gene, active under both limiting conditions and by induction, allowing a high degree of freedom in its regulation and tunability. Furthermore, we present our results with respect to the characterization of a novel bi-directional promoter system, where we combined this novel P_DC_ variant and P_AOX1_ in a bi-directional manner. In this feasibility study, we cloned two gene variants of the model enzyme cellobiohydrolase 2 (CBH2) downstream of either promoter to demonstrate the proof-of-concept that the expression level of this product can solely be tuned by induction conditions using the bi-directional promoter system. We used the same product downstream of the two promoters to exclude the possibility that different products are differently well expressed due to product-specific features, not only affecting productivity but also strain physiology. Thus, we ensured that the effects on productivity and physiology only resulted from induction conditions and the consequent regulation of the two promoters and not from product-specific features. In summary, our results nicely demonstrate tunability of a novel bi-directional promoter system solely in response to cultivation conditions, which widens the toolbox for *P. pastoris*. A potential future application of this system could be the subsequent production a chaperone and a target protein, as schematically shown in Fig. 1.

## Methods

### Host organism and model protein

A *P. pastoris* BSYBG11 strain harbouring a novel bi-directional promoter system, comprising the modified derepressible and inducible P_DC_ and the methanol inducible P_AOX1_, was constructed by Bisy e.U. (Hofstaetten/Raab, Austria). BSYBG11 is a killer-plasmid-free next generation platform strain, based on the NRLY 11430 wildtype strain, where the AOX1 gene was deleted according to Sturmberger et al. [27]. The lignocellulolytic enzyme CBH2 derived from *Trichoderma reesei* [28] was used as a model protein in this study and cloned downstream of either promoter. Furthermore, the strain carried a Zeocin resistance gene, and the pre-pro signal sequence of the alpha-mating factor allowed product secretion.

### Bioreactor cultivations

All bioreactor cultivations were carried out in a 5 L lab scale glass bioreactor (Infors, Switzerland). Monitoring and control of process parameters was done by the Lucullus process information management system (PIMS; Biospectra, Switzerland). pH was controlled at pH 5.0 and temperature at 30 °C. The dissolved oxygen concentration (dO_2_) was kept above 30% with a cascaded control of agitation and addition of pure oxygen.

Strain specific physiological parameters, such as substrate uptake rates (q_s_) and biomass yields (Y_X/S_), are prerequisites for designing efficient fed-batch strategies. Thus, dynamic cultivations with shifts in q_s_ and repeated substrate pulses were done to evaluate these strain specific physiological parameters, according to our previous studies (e.g. [29–32]). Based thereon, mixed feed cultivations were carried out to analyze tunability of recombinant protein production. A summary of the three dynamic cultivations performed in this study and their respective goals is shown in Table 1.

**Table 1:** Dynamic cultivations performed with the recombinant *P. pastoris* strain carrying a novel bi-directional promoter system

Experiment	Substrate	Feeding strategy based on q_s_ [g g^−1^ h^−1^]^a^	Goals
FB1	Glycerol	Batch on glycerol Fed-batch on glycerol with step-wise decrease of q_s,gly_ 0.567–0.450–0.282–0.142–0.101–0.070–0.050 g g^−1^ h^−1^ (each step was done for 2 h) Methanol pulses: 0.5% (v/v) adaptation pulse at 30 °C—two 2% (v/v) pulses at 30 and 20 °C, respectively	Determination of strain specific physiological parameters (q_s,max,gly_; q_s,gly_ for maximum q_p_; q_s,adapt,MeOH_; q_s,max,MeOH_) Characterization of recombinant expression profile at different process parameters
FB2	Glycerol and methanol	Batch on glycerol 0.5% (v/v) methanol adaptation pulse Constant MeOH feed at q_s_ 0.02–0.025 g g^−1^ h^−1^ and concomitant glycerol feed with stepwise increases: 0.025-0.059-0.139-0.239 g g^−1^ h^−1^ (each step was done for 2 h)	Find operating window for maximum q_p_
FB3	Glycerol and methanol	Batch on glycerol Fed-batch at q_s,gly_ = 0.212 g g^−1^ h^−1^ 0.5% (v/v) methanol adaptation pulse followed by a fed-batch on methanol at q_s_ = 0.034 g g^−1^ h^−1^ Mixed feed cultivation at q_s,gly_ = 0.135 g g^−1^ h^−1^ and q_s,MeOH_ = 0.035 g g^−1^ h^−1^	Demonstrate tunability of recombinant protein production

^a^The q_s_ values were adjusted based on our findings during this study (vide infra)

Prior to all cultivations, precultures were prepared with frozen cryo-stocks in yeast nitrogen base medium (YNB) supplemented with Zeocin. The preculture was incubated at 30 °C and 230 rpm overnight. The batch phase for all cultivations was started by adding the preculture to the bioreactor [10% (v/v)]. After the batch, different feeding strategies were applied (Table 1). All cultivations were carried out in twofold basal salt medium (BSM) with a final glycerol concentration of 60 g L^−1^ in the batch medium. Samples were taken throughout the cultivations for offline analyses. In all the cultivations, the feed rate (FR) was calculated from biomass (c_x_) and substrate (c_s_) concentrations, volume of the bioreactor (V_R_) and specific substrate uptake rates (q_s_) and controlled using a feed forward strategy (Eq. 1).

1 $$\text{FR}_{\text{t}} \text{ = }\frac{{\text{c}_{\text{X}} \cdot \text{V}_{\text{R}} }}{{\text{c}_{\text{S}} }} \cdot \text{q}_{\text{S}}$$

### Offline data analysis

Biomass concentration was determined by optical density measurements at 600 nm and dry cell weight measurement, as described before (e.g. [29–32]). Protein concentration was measured at 595 nm by the Bradford assay using the Sigma-Aldrich protein assay kit with bovine serum albumin as standard in the range of 0.2–1.2 mg mL^−1^. The specific productivity (q_p_) was determined from the total extracellular protein concentration measured in the cell-free cultivation broth and the respective dry cell weight. Product formation and electrophoretic purity was checked by SDS-PAGE.

## Results and discussion

In this study, a recombinant *P. pastoris* strain harbouring a novel bi-directional promoter system with the two promoters P_DC_/P_AOX1_ was characterized and analyzed for tunable recombinant production of the model enzyme CBH2. The goal of this proof-of-concept study was to show that expression can be stirred solely by induction conditions using this novel bi-directional system, which is why we used the same model product to exclude product-dependent variations in productivity as well as product-dependent effects on physiology. We performed three dynamic bioreactor cultivations to characterize the recombinant *P. pastoris* strain (Table 1). In all of these cultivations we determined a maximum specific growth rate (µ_max_) on glycerol of 0.27 h^−1^, a q_s,max,gly_ of 0.57 g g^−1^ h^−1^ and a biomass yield (Y_X/S_) on glycerol of 0.47 g g^−1^. These values compare well with values we found for a Mut^S^ benchmark strain before [33], indicating that the introduced genetic construct had no negative impact on the physiology of the *P. pastoris* strain.

### Fed-batch 1 (FB1)

In the first dynamic fed-batch, we determined strain specific physiological parameters and characterized the recombinant expression profile at different process parameters (Table 1). After complete consumption of glycerol in the batch phase, we stepwise decreased q_s,gly_ in the subsequent fed-batch to find q_s,gly_ where the P_DC_ promoter was fully active. We determined the specific productivity (q_p_) as a measure for promoter activity. As shown in Table 2, the P_DC_ promoter was actually never repressed, not even at high q_s,gly_. However, we found an optimum in q_p_ at a q_s,gly_ of 0.28 g g^−1^ h^−1^. At lower q_s,gly_, q_p_ decreased again, as the cells came close to their maintenance metabolism, which was also visible in the specific yields (Table 2). Closing C-balances underlined the validity of the calculated physiological strain specific parameters.

**Table 2:** Dynamic glycerol fed-batch phase to characterize the P_DC_ in FB 1

Steps	q_s,gly_ (g g^−1^ h^−1^)	µ (h^−1^)	q_p_ (mg g^−1^ h^−1^)	${\text{Y}}_{{{{{\text{CO}}_{2} } \mathord{\left/ {\vphantom {{{\text{CO}}_{2} } {\text{S}}}} \right. \kern-0pt} {\text{S}}}}}$ (Cmol Cmol^−1^)	Y_X/S_ (Cmol Cmol^−1^)	C-balance (−)
1	0.567	0.318	0.307	0.28	0.67	0.95
2	0.450	0.251	0.362	0.34	0.67	1.01
3	0.282	0.132	0.492	0.37	0.56	0.92
4	0.142	0.068	0.349	0.44	0.51	0.95
5	0.101	0.056	0.124	0.46	0.47	0.93
6	0.070	0.039	0.121	0.51	0.43	0.94
7	0.050	0.023	0.073	0.58	0.37	0.95

After the dynamic glycerol phase in FB1, we added a 0.5% (v/v) methanol adaptation pulse, followed by two 2% (v/v) pulses at each 30 and 20 °C, respectively, to analyze the adaptation characteristics to methanol and determine the specific uptake rate of methanol (q_s,MeOH_) as well as q_p_ at both temperatures (Table 3).

**Table 3:** Methanol pulses at 30 and 20 °C, respectively, to characterize the P_DC_ in FB1

Phases	q_s,MeOH_ (g g^−1^ h^−1^)	q_P_ (mg g^−1^ h^−1^)
0.5% adapt	0.011	0.021
2% MeOH_T=30 °C_	0.035	0.115
2% MeOH_T=20 °C_	0.039	0.056

The adaptation time, which is the time taken for adaptation of the culture to the new substrate methanol [29, 30], of the strain carrying the bi-directional promoter system to methanol at 30 °C was only 3 h, which was much lower compared to a Mut^S^ benchmark strain, where we had found adaptation times of more than 6 h [29, 33]. This drastic reduction might be due to the concomitant presence of two promoters, which are inducible by methanol.

As shown in Table 3, the specific uptake rate of methanol was higher at 20 °C than at 30 °C. However, q_p_ was higher at 30 °C, which is why we chose this temperature for the subsequent fed-batch phases on methanol.

### Fed-batch 2 (FB2)

In FB2, we adapted one of our previous mixed feed strategies to allow fast physiological strain characterisation as well as bioprocess development in a mixed feed environment [34]. We constantly fed methanol at a q_s_ = 0.022–0.025 g g^−1^ h^−1^, corresponding to around 75–80% of q_s,max,MeOH_ at 30 °C (Table 3), and concomitantly fed glycerol, which we stepwise increased (Table 1). In the different phases, we determined specific rates and yields, to demonstrate tunability of the system and to find a good operating window allowing highest q_p_ (Table 4). Again, closing C-balances underlined the validity of the calculated strain specific parameters.

**Table 4:** Dynamics in FB2 to analyze tunability and to find an operating window for the bi-directional promoter system allowing highest q_p_

Steps	q_s,gly_ (g g^−1^ h^−1^)	q_s,MeOH_ (g g^−1^ h^−1^)	µ (h^−1^)	q_p_ (mg g^−1^ h^−1^)	${\text{Y}}_{{{{{\text{CO}}_{2} } \mathord{\left/ {\vphantom {{{\text{CO}}_{2} } {\text{S}}}} \right. \kern-0pt} {\text{S}}}}}$ (Cmol Cmol^−1^)	Y_X/S_ (Cmol Cmol^−1^)	C-balance (−)
1	0.025	0.022	0.005	0.109	0.78	0.14	0.92
2	0.059	0.024	0.009	0.372	0.71	0.23	0.94
3	0.139	0.025	0.031	0.349	0.55	0.42	0.97
4	0.239	0.023	0.112	0.099	0.42	0.57	0.99

As shown in Table 4, q_p_ could be tuned by adjusting different q_s_ ratios. Interestingly, the highest q_p_ was achieved in the presence of methanol at a rather low q_s,gly_. Compared to the q_p_ at a similar q_s,gly_ in FB1, which was 0.073 mg g^−1^ h^−1^ at q_s,gly_ = 0.050 g g^−1^ h^−1^, we obtained a more than fivefold higher q_p_ of 0.372 mg g^−1^ h^−1^ at q_s,gly_ = 0.059 g g^−1^ h^−1^ in the presence of methanol. Furthermore, we found a 1.5-fold higher q_p_ of 0.115 mg g^−1^ h^−1^ compared to the sole presence of methanol (Table 3). Increasing q_s,gly_ in the presence of methanol had a negative impact on q_p_, which we ascribe to repression effects, as reported in similar studies before [34]. Offline analysis confirmed no accumulation of methanol in the mixed-feed phase.

### Fed-batch 3 (FB3)

In the final cultivation (FB3), we demonstrated tunability of recombinant protein production by adjusting different feeding regimes. We analyzed productivity as well as the the strain specific, physiological parameters on glycerol, on methanol as well as in a mixed feed environment (Table 1). The results are summarized in Table 5.

**Table 5:** Strain specific, physiological parameters determined in FB3

Phase	q_s,gly_ (g g^−1^ h^−1^)	q_s,MeOH_ (g g^−1^ h^−1^)	µ (h^−1^)	q_p_ (mg g^−1^ h^−1^)	${\text{Y}}_{{{{{\text{CO}}_{2} } \mathord{\left/ {\vphantom {{{\text{CO}}_{2} } {\text{S}}}} \right. \kern-0pt} {\text{S}}}}}$ (Cmol Cmol^−1^)	Y_X/S_ (Cmol Cmol^−1^)	C-balance (−)
Glycerol	0.212	–	0.122	0.368	0.40	0.57	0.97
Methanol	–	0.034	0.003	0.097	0.83	0.15	0.98
Mixed feed	0.135	0.035	0.106	0.573	0.54	0.41	0.95

**Fig. 2:**
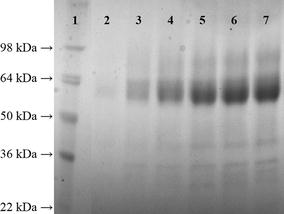
SDS-PAGE gel of cell-free cultivation broth taken at different time points during FB3. Lane 1, protein ladder; lane 2, sample after batch on glycerol; lanes 3–5, samples during fed-batch at q_s,gly_ = 0.212 g g^−1^ h^−1^ (samples were taken every 2 h); lane 6, sample after methanol fed-batch; lane 7, sample after mixed feed phase. Size CBH2 = ca. 60 kDa

The q_p_ in the glycerol fed-batch phase was comparable to the results we obtained in FB1 (Table 1). At a q_s,gly_ = 0.212 g g^−1^ h^−1^ we calculated a q_p_ = 0.368 mg g^−1^ h^−1^. In the subsequent fed-batch phase on methanol, where we adjusted a q_s,MeOH_ = 0.034 g g^−1^ h^−1^, which was close to q_s,max,MeOH_, we calculated a q_p_ of 0.097 mg g^−1^ h^−1^. When we concomitantly fed glycerol at a q_s,gly_ = 0.135 g g^−1^ h^−1^, we were able to boost q_p_ to 0.573 mg g^−1^ h^−1^, which was the highest value we obtained in all the experiments performed in this study. This value was higher than the q_p_ we achieved in the mixed feed environment at q_s,gly_ = 0.139 g g^−1^ h^−1^ in FB2, probably due to a higher q_s,MeOH_. Again, no methanol accumulation was identified during this cultivation. Closing C-balances underlined the validity of the calculated strain specific parameters. We analyzed the cell-free cultivation broth at different time points of the cultivation on SDS-PAGE gels. As shown in Fig. 2, the main protein fraction in the cell-free cultivation broth constituted for the recombinant product CHB2, which is why it was legitimate to use the total extracellular protein content to calculate productivities.

## Conclusions

In this study we present the first controlled bioreactor experiments with a novel bi-directional promotor combination of a catalase promoter variant (P_DC_), which shows high activity in the presence of glycerol, but is also inducible by methanol, and the traditional P_AOX1_. By performing dynamic bioreactor cultivations, we physiologically characterized the recombinant strain and determined conditions allowing high productivity of the strain in only three experiments. Finally, we demonstrated that this bi-directional promoter system allows tunable recombinant protein expression solely in response to the available C-sources. This bi-directional promoter system offers a high degree of freedom for bioprocess design and development making bi-directional promoters in *P. pastoris* highly attractive for recombinant protein production.

## Abbreviations

CBH2: cellobiohydrolase 2
q_P_: strain specific productivity [mg g^−1^ h^−1^]
q_S_: strain specific substrate uptake rate [g g^−1^ h^−1^]
P_DC_: catalase promoter variant
P_AOX1_: alcohol oxidase promoter
P_GAP_: glyceraldehyde-3-phosphate dehydrogenase promoter
PIMS: process information management system
dO_2_: dissolved oxygen concentration [%]
Y_X/S_: yield of biomass per substrate [Cmol Cmol^−1^]
YNB: yeast nitrogen base
BSM: basal salt media
F_R_: feed rate [L h^−1^]
c_S_: concentration of substrate [g L^−1^]
c_X_: biomass concentration [g L^−1^]
V_R_: volume of the reactor [L]
${\text{Y}}_{{{{{\text{CO}}_{ 2} } \mathord{\left/ {\vphantom {{{\text{CO}}_{ 2} } {\text{S}}}} \right. \kern-0pt} {\text{S}}}}}$: yield of carbon dioxide per substrate [Cmol Cmol^−1^]
